# Minimally invasive techniques for sinus floor elevation using dental implants among Indians

**DOI:** 10.6026/973206300191336

**Published:** 2023-12-31

**Authors:** Shyamalendu Laskar, Deepmala Pande, Md. Shadab Anwar, Akshayraj Langaliya, Ruchira Shukla, Harshit Shrivastava, Dhaval Niranjan Mehta, Sheetal Acharya

**Affiliations:** 1 Department of Oral and Maxillofacial Surgery, Agartala, Tripura, India; 2 Department of Prosthodontics and Crown and Bridge, Hitkarni Dental College and Hospital, Jabalpur, M.P., India; 3 Department of Periodontology, Mithila Minority Dental College and Hospital, Darbhanga, Bihar, India; 4 Department of Conservative Dentistry and Endodontics, AMC Dental College and Hospital, Gujarat, India; 5 Department of Oral and Maxillofacial Surgery, Ambikapur, Chhattisgarh, India; 6 Department of Oral and Maxillofacial Surgery, Hitkarni Dental College and Hospital, Jabalpur, M.P, India; 7 Department of Oral Medicine and Radiology, Narsinbhai Patel Dental college and Hospital, Sankalchand Patel University, Visnagar, Gujarat, India; 8 Department of Periodontics and Implantology, Kalinga Institute of Dental Sciences KIIT (Deemed to be) University, Bhubaneswar, India

**Keywords:** Sinus augmentation, conventional techniques, minimally invasive techniques, dental implants

## Abstract

It is of interest to compare conventional lateral window technique, trans-alveolar technique with minimally invasive technique like modified trephine technique and antral membrane balloon elevation technique for sinus floor elevation in placement of dental implants. The current study included 140 participants (191 dental implants) who had maxillary posterior edentulous regions and had low sinuses and insufficient alveolar ridges but had chosen an implant-retained prosthesis. The minimally invasive techniques of sinus augmentation can be suitable alternative to conventional traumatic techniques due to low incidence of pain, gingival swelling. Moreover, the success rate of implants and increase in total bone height was almost same in minimally invasive techniques of sinus augmentation as compared to conventional traumatic techniques.

## Background:

Given the phenomenon of maxillary sinus (MS) pneumatization, placing endosseous implants in the posterior tooth-free maxilla is typically a difficult procedure in implant dentistry [1-2]. With remarkable success rates, a variety of sinus augmentation procedures have been employed to prepare these areas for implant implantation [3-4]. Understanding the MS anatomy aids in both appropriate preoperative treatment-planning, and preventing potential complications during MS augmentation procedures [5-6]. Prior to placing dental implants, MS augmentation, also called sinus floor elevation, has grown in popularity in upper back maxillae that have undergone significant bone loss from trauma, sinus pneumatization, or alveolar bone atrophy [7-8, 9]. By applying graft material, Hilt Tatum increased the amount of available bone in MS space in the 1970s, allowing for a larger implant to bone contact area after the bone graft matured [10- 11,12]. A surgeon's choice and the physical characteristics of the patient will determine the method of MS elevation along with augmentation that is used on a particular patient [13- 14]. The intended quantity of lift and the residual bone dimensions are examples of patient anatomy. The direct and indirect techniques constitute the two main methods for elevating the MS floor [15- 16]. The least-invasive trans-alveolar sinus approach, antral membrane balloon elevation, osteotome (MS) floor elevation, and bone added MS floor elevation are examples of indirect methods while lateral window methodology constitute the direct technique [17,18]. By means of a window made in the MS lateral wall, the sinus membrane is directly seen and configured in the direct/lateral window technique [19-20]. The primary disadvantage of lateral antrostomy is the need to elevate a sizable flap in order to gain surgical access. This method takes longer and is more technique-sensitive. The dimensions of remaining bone are the primary determinant of the procedure's success [21- 22].

It is generally recommended to use the indirect, osteotome, crestal, or trans-alveolar approaches when the remaining bone height is 6 mm or greater. After the graft material is in place, this is accomplished by re-entering the largest osteotome into the implant site. The sinus membrane is pressed upon by the additional bone graft, raising it even higher [23- 24]. To raise MS membrane to the required level, bone graft might be added as well as tapped. Don't raise the membrane beyond its breaking point [25-26]. Using a direct or osteome approach of raising the sinus floor raises the likelihood of a sinus membrane perforation. Consequently, over time, a few minimally invasive methods of sinus floor augmentation are being developed [16]. The modified trephine/osteotome technique has been introduced where a 3 mm exterior diameter trephine bur is used to prepare the area for implant placement, keeping it 1-2 mm from the MS floor [18].

By applying an osteotome with comparable diameter as the trephine bur, the bone cylinder is subsequently pushed apically to a depth that is 1 mm shorter than the one created with the bur [16,18]. Osteotomes of varying diameters are used to complete the preparation of the area for implant placement, frequently placing them to equivalent depth. The bone cylinder inside the region resulting from the shifting of the sinus membrane experiences regulated lateral movement as a result of the implants being inserted at an average rate of 30 revolutions per minute [22]. It has been demonstrated that this method lowers the risk of sinus membrane perforation [22]. There is need to evaluate the effectiveness of these minimally invasive techniques when compared to direct or indirect surgical technique for sinus elevation. It will help to understand whether these minimally invasive techniques can be used as an alternative to traumatic direct and indirect surgical approaches of sinus floor elevation. Therefore, it is of interest to compare conventional lateral window technique, trans-alveolar technique with minimally invasive technique like modified trephine technique and antral membrane balloon elevation technique for sinus floor elevation in placement of dental implants.

## Methods and Materials:

The current study included 140 participants (191 dental implants) who had maxillary posterior edentulous regions and had low sinuses and insufficient alveolar ridges but had chosen an implant-retained prosthesis. Exclusions from the study included smokers, pregnant women, patients with long-term nasal obstruction, patients who have on-going sinusitis, and patients with psychological illnesses.

Using CBCT, the maxillary sinus was assessed in accordance with the Misch criteria [14].

[1] SA-1 site with residual ridge remaining 12 mm or more.

[2] The SA-2 site has 10 to 12 mm of remaining alveolar ridge

[3] The SA-3 site has a minimum of 5 mm of remaining alveolar ridge

[4] The SA-4 site has less than 5 mm of remaining alveolar ridge

Commercially available pure titanium implants with an appropriate taper were utilised. The implant measured 8mm, 10mm, 11,5mm, 13mm, and 16 mm in length and 3.3mm, 3.75mm, 4.2mm, and 5 mm in diameter. The preferred graft utilised for this research was a xeno-graft called Bio-Oss (Geistlich Biomaterials, Switzerland). This xeno-graft is non-immunogenic and most likely protected from infection risk because all organic material has been eliminated, leaving only the mineralized bone architecture.

Both medication and local anesthetic were used during the surgical operations. Four distinct categories of patients-category 1 (direct sinus lift), category 2 (trans-alveolar sinus lift), category 3 (modified trephination technique), and category 4 (antral ballon sinus elevation)-were randomly and equally assigned (Table 1). All patients began preoperative antibiotic therapy one day prior to surgery, taking 625 mg of clavulanic acid and amoxicillin three times a day.

## Surgical procedure:

## Category 1:

A small incision was made extending anteriorly from the canine eminence and posteriorly upto zygomatic buttress, directly above the muco-gingival intersection. Using a mallet and 4 mm chisel and 6 mm chisels, a rectangular window was made in the canine fossa once a mucoperiosteal flap was raised from the buccally and superiorly incision. The anterior osteomy cut, posterior osteomy cut, and superior osteotomy cuts were made after the inferior osteotomy cut, which was made approximately 4-5 mm above the maxillary sinus floor. The size of the designed osteotomy was somewhere 1 x 1 cm, which was adequate to provide convenient access for simple dissection, elevation of the sinus membrane, and graft insertion. Commencing from the inferior cuts and lateral cuts, the MS membrane was separated from the underlying bone without rupturing, releasing enough mucosa to permit tension-free reflection from the sinus floor. The process of dissection was continued until osteomy window could be reflected inward and superiorly to the required height. In none of the cases was there a sinus membrane perforation. After extracting 5 millilitres of whole blood from the patient's antecubital fossa, the graft material (Bio-Oss) was opened and put into a dish. The graft was then combined with an adequate volume of whole blood. The elevated MS membrane was lifted after exposing the osteotomy site. After obtaining sufficient elevation, the study participants whole blood mixed with particulate graft were packed into the sinus cavity. Above the grafted site, a collagen barrier membrane was applied. 4-0 silk was used to seal the incision.

## Category 2:

The incision was made long enough to reveal every implant site, from the palatal crest to the alveolar crest. To provide sufficient tension-free buccal reflection of the soft tissue flap, two vertical releasing incisions have been established at the anterior extent and posterior extent of the initial incision. The mucoperiosteal flap was raised from the incision, being cautious not to puncture it at the alveolar crest, both buccally as well as superiorly. Internal irrigation was utilised for bone drilling during the antrostomy, which was carried out using a hand piece with a speed reduction gear. To prepare the site, a series of surgical twist drills with diameters that ranged from 2.0mm to 4.8 mm were employed.

Using a series of sinus osteotomes and a metal mallet, the palatal osseous lid was entirely removed, and the MS membrane was painstakingly dissected and raised. Following total elevation in each case, an organic bovine bone graft was used to reconstruct the sinus cavity. The biomaterial was packed tightly into the cavity after being combined with blood drawn from the study participant's ante-cubital fossa. There were no more autogenous bone chips or blocks utilised. The implant of the chosen size was inserted once the entire prepared space had been filled. The fixture insertion instrument was engaged with the implant, the implant holder was dragged, and light pressure was applied.

The implant was firmly screwed into the bone using a hex ratchet until all of its sides lined up with the alveolar bone crest. The graft material's surplus particles were eliminated, and the palatal flaps were moved without making single periosteal horizontal releasing cuts. A 4-0 silk suture was used to close the wound.

## Category 3:

By applying an osteotome with comparable diameter as the trephine bur, the bone cylinder is subsequently pushed apically to a depth that is 1 mm shorter than the one created with the bur. Osteotomes of varying diameters are used to complete the preparation of the area for implant placement, frequently placing them to equivalent depth. The bone cylinder inside the region resulting from the shifting of the sinus membrane experiences regulated lateral movement as a result of the implants being inserted at an average rate of 30 revolutions per minute.

## Category 4:

Soltan *et al.* [18] described the antral membrane balloon elevation method. This method elevates the sinus membrane by using an inflatable balloon. The goal of the Zimmer MS augmentation balloon is to evenly and gently elevate the sinus membrane. Following surgery, all patients in category 1, category 2, category 3 and category 4 received the same prescription (625 mg of amoxyclav), along with three daily doses of metronidazole 400 mg, a five-day course of aciclofenac 100 mg, 500 mg of paracetamol, and a nasal decongestant. The standard postoperative instructions for the patients were to rinse well with antimicrobial mouthwash (chlorhexidine gluconate 0.2%), apply an ice pack, and maintain a soft, high-nutrient diet. The patients were advised not to inhale deeply or produce a vacuum or high intranasal pressure by blowing their noses or sneezing. For a week, the patients were told not to drink through straws. To lower the risk of wound dehiscence, the patients were told not to use any prosthesis over the area of operation for at least one week following the procedure. Patients in both groups underwent post-operative follow-up at one week, three weeks, six weeks, and twelve weeks. Additionally, follow-up was conducted at one, two, and three months following implant insertion to check implant stability, pain, gingival inflammation.

## Measurements:

CBCT radiographs and a clinical examination were used to determine measurements. For every group, information was gathered on the number of implants per patient, the location of implants, and the width and length of implants. Measures of RBH prior to surgery and total increase in bone height following completion of all treatments were performed on CBCT images for four groups. All surgical groups had their graft healing times and increase in bone height from first sinus lifting before implant placement measured.

## Statistical analysis:

Excel (Microsoft, Seattle, WA, USA) and SPSS (Version 17.0.1, Armonk, NY, USA) were the statistical software packages used for the analyses. The means and standard deviations (SD) of the implant characteristics for each of the four treatment groups were computed as descriptive statistics. The Chi-Squared test was used to examine the implant length and width distribution for each group. Following the initial stage of surgery, all categories were compared for variations in time for healing of graft and the increase in height of bone of first sinus lifting using the Mann-Whitney U test. The differences in RBH, complete increase in height of bone, and time period of surgical rehabilitation between the four groups were assessed using the Kruskal-Wallis test. P<.05 was chosen as the significance threshold for all statistical tests.

## Results:

The most common location for implant placement in all categories was molar region. The most common length of dental implants placed in all categories was 10mm and 11.5 mm (Table 2 and Table 3). The pre-treatment RBH was 2.89±0.56 mm, 2.27±0.84 mm, 2.38±1.25 mm and 1.39 ±0.88 mm in category 1,2,3 and 4 respectively. The total increase in bone height after 12 months follow up was 12.96±2.22 mm, 11.66±1.67mm, 10.94±1.60mm and 8.42±1.21mm respectively in category 1,2,3 and 4. It was observed that there was statistically significant increase in bone height in all study participants after complete treatment. However, when there was comparison between different categories showed no significant variations in increase in bone height among them (Table 4). On evaluating pain through VAS score and gingival swelling, the intensity of pain and gingival swelling felt by patients in category 3 (modified trephination technique) and category 4 (antral balloon technique) was lesser as compared to category 1 (direct lateral window technique) and category 2 (trans=alveolar technique). The average stability of implants in all categories was about 98.9%, 97.23%, 94.4% and 96.3% respectively. In our study there was complete success in all implants except one implant.

## Discussion:

There is need to evaluate the effectiveness of these minimally invasive techniques when compared to direct or indirect surgical technique for sinus elevation. It will help to understand whether these minimally invasive techniques can be used as an alternative to traumatic direct and indirect surgical approaches of sinus floor elevation. Therefore this study was carried out to compare conventional lateral window technique, trans-alveolar technique with minimally invasive technique like modified trephine technique and antral membrane balloon elevation technique for sinus floor elevation in placement of dental implants. According to earlier research, patients with an RBH of at least 5 mm should only undergo sinus augmentation with Trans-alveolar bone-added osteotome sinus floor elevation (BAOSFE); patients with an RBH of less than 4 mm should undergo lateral window sinus lift [26, 27,28,29]. Data shows that if a one stage or two stage technique was employed, the rate of successful placement of the implant employing BAOSFE was higher while the RBH was ≥ 5 mm [15-17]. Implant longevity rates with a lateral window sinus lift were beneficially correlated when the RBH was ≥5 mm, according to a meta-regression study examining the connection between the RBH and the survival of implants complying with lateral window or osteotome MS elevation strategies as described elsewhere [14-18]. For trans-alveolar MS lift techniques, however, no relationship could be found because there was insufficient data in the included studies to support an initial RBH of less than 4 mm [12-18].

A more recent meta-analysis by revealed that an initial RBH of <4 mm was positively correlated with implants inserted in conjunction with trans-alveolar MS elevation techniques, but it had no effect on implant success or failure [16- 20]. It has been demonstrated that a lateral window sinus lift approach can increase bone height without being constrained by the dimension of the pre-operative RBH [14-17]. All except one of the implants in the current study, which ranged in length from 8.5 to 13 mm, were successful. These results contrast with those indicating a lower success rate associated with shorter implants. An infrabony length that was less than 8 mm was the criteria to classify short implants [18-21]. Conversely, because a longer length allows for a more effective transportation of functional forces all through the implants, standard implants ≥10 mm have a high predictability [19-20]. Comparable survival rates have been found in more recent studies for implants of standard length and shorter length. For example, survival rates varied between 92.2-100% in a systematic examination of 17 studies with short implants (<8 mm) and observation times spanning 3 months to 9 years [21-26].

The survival rates of short as well as long implants did not differ significantly, according to a systematic review of 33 studies [23-27]. But a examined five randomized controlled clinical trials with a follow-up period of 16 to 18 months, and they contrasted shorter implants (<8 mm) in the posterior maxilla with longer implants (>8 mm) positioned concurrently or following trans-alveolar or lateral window MS elevation techniques. The survival rates (99.5 percent and 99.0 percent, respectively) for shorter as well as longer implants were comparable. However, longer dental implants placed in the augmented sinus had more complications (nearly three times as many), mostly from surgically caused membrane perforations. Regardless of implant length, none of the dental implants in our study had membrane perforations. The length of the failed implant was 10 mm, and the absence of infection indicates that osseointegration failure was probably the cause of the loss [21-24]. According to our research, a pre-operative measurement of <3 mm for RBH may be significant for figuring out what amount of bone gain can be obtained but is not always indicative of implant failure. Consequently, when determining implant length in relation to the required amount of bone gain, the starting point RBH should be taken into account.

Some studies also showed minimum swelling and pain after minimally invasive techniques for sinus elevation [18, 22]. A study noted a gradual decrease in pain following sinus lift surgery, but they also showed two patients experiencing pain associated with sinusitis, which they determined was caused by the sequestrum of the cancellous bone migrating into the maxillary sinus [18-20]. For this patient, they conducted a sinuscopy and removed the sequestrum. Our data showing little pain after surgery are consistent with theirs.

Another study showed similar data [12-16]. According to them, patients with preoperative maxillary sinus disease experience postoperative complications like pain, swelling, disrupted wound healing, temporary maxillary sinusitis, and implant failures. In contrast, non-significant post-operative swelling was noted in otherwise healthy patients, which is consistent with our data. We observed excellent soft-tissue response and rapid healing in our study, which is consistent as reported elsewhere. Lateral antrostomy needed a larger surgical incision but allowed for more bone augmentation to be added to the atrophic maxilla. The crestal approach is extremely minimally invasive but only allows for a restricted amount of augmentation as shown elsewhere [17-22].

Current data is consistent with known study, which demonstrated that implants inserted utilising three distinct sinus augmentation techniques were successful and had comparable survival rates following a minimum three-year observation period. Our data shows that implant survival rates in trans-alveolar sinus floor augmentation sites are on par with non-augmented site survival rates. This method has a low rate of complications both during and after surgery, making it predictable as shown elsewhere [24].

## Conclusion:

The minimally invasive techniques of sinus augmentation are a suitable alternative to conventional traumatic techniques due to low incidence of pain and gingival swelling. Moreover, the success rate of implants and increase in total bone height was almost same in minimally invasive techniques of sinus augmentation as compared to conventional traumatic techniques.

## Figures and Tables

**Table 1:** Distribution of study participants in different categories

	**Technique used**	**No of study participants (n)**	**Total no of dental implants**
Category 1	Lateral window technique (direct)	34	46
Category 2	Transalveolar technique	36	48
Category 3	Modified trephination tevhnique	33	48
Category 4	Antral balloon elevation	37	49

**Table 2:** Position of implants in different categories (n (%)

	**Pre-molar**	**Molar**
Category 1	10 (23.8)	34 (78.4)
Category 2	08 (14.9)	48 (86.8)
Category 3	10(22.8)	36 (79.4)
Category 4	09 (24.6)	36 (79.7)
P value	0.605	

**Table 3:** Implant length in four categories (n(%))

	**8.5 mm**	**10.0 mm**	**11.5 mm**	**13.0 mm**	**P value**
Category 1	28(64.5)	14 (32.9)	2 (4.6)	0 (0.0)	
Category 2	7 (11.8)	18 (33.2)	32 (58.2)	0 (0.0)	0.004
Category 3	2 (5.4)	16 (35.8)	22 (48.9)	6 (13.1)	
Category 4	1(1.6)	26 (57.8)	18 (40.2)	2 (8.7)	

**Table 4:** Measurements (mean±SD)

	**RBH, pre-treatment, mm**	**Graft healing time, months**	**Total bone height gain, mm**
Category 1	2.89±0.56	7.60± 2.00	12.96±2.22
Category 2	2.27±0.84	9.84±2.22	11.66±1.67
Category 3	2.38±1.25	-	10.94±1.60
Category 4	1.39 ±0.88	-	8.42±1.21
P value		0.004	
